# Improvement in Heat Transfer in Hydrocarbon and Geothermal Energy Coproduction Systems Using Carbon Quantum Dots: An Experimental and Modeling Approach

**DOI:** 10.3390/nano15120879

**Published:** 2025-06-07

**Authors:** Yurany Villada, Lady J. Giraldo, Diana Estenoz, Masoud Riazi, Juan Ordoñez, Esteban A. Taborda, Marlon Bastidas, Camilo A. Franco, Farid B. Cortés

**Affiliations:** 1 Grupo de Investigación Fenómenos de Superficie Michael Polanyi, Departamento de Procesos y Energía, Facultad de Minas, Universidad Nacional de Colombia, Sede Medellín, Medellín 050034, Colombia; yavillad@unal.edu.co (Y.V.); ljgiraldop@unal.edu.co (L.J.G.); eatabord@unal.edu.co (E.A.T.); 2 Instituto de Desarrollo Tecnológico para la Industria Química (INTEC), Consejo Nacional de Investigaciones Científicas y Técnicas (CONICET), Universidad Nacional del Litoral, Güemes 3450, Santa Fe 3000, Argentina; destenoz@santafe-conicet.gov.ar; 3 School of Mining and Geosciences, Nazarbayev University, Astana 010000, Kazakhstan; masoud.riazi@nu.edu.kz; 4 Center for Advanced Power Systems, Department of Mechanical Engineering, FAMU-FSU College of Engineering, Energy and Sustainability Center, Florida A&M University-Florida State University, Tallahassee, FL 32310, USA; ordonez@eng.famu.fsu.edu; 5 Grupo Destacar, Universidad de La Guajira, Km 5, vía Maicao, Riohacha 440002, Colombia; marlonjoseb@uniguajira.edu.co

**Keywords:** carbon quantum dots, coproduction, decarbonization, geothermal energy, thermal conductivity, renewable energy, simulation

## Abstract

The main objective of this study is to improve heat transfer in hydrocarbon- and geothermal-energy coproduction systems using carbon quantum dots (CQDs). Two types of 0D nanoparticles (synthesized and commercial CQDs) were used for the formulation of nanofluids to increase the heat transfer from depleted wells for the coproduction of oil and electrical energy. The synthesized and commercial CQDs were characterized in terms of their morphology, zeta potential, density, size, and heat capacity. The nanofluids were prepared using brine from an oil well of interest and two types of CQDs. The effect of the CQDs on the thermophysical properties of the nanofluids was evaluated based on their thermal conductivity. In addition, a mathematical model based on heat transfer principles to predict the effect of nanofluids on the efficiency of the organic Rankine cycle (ORC) was implemented. The synthesized and commercial CQDs had particle sizes of 25 and 16 nm, respectively. Similarly, zeta potential values of 36 and 48 mV were obtained. Both CQDs have similar functional groups and UV absorption, and the fluorescence spectra show that the study CQDs have a maximum excitation–emission signal around 360–460 nm. The characterization of the nanofluids showed that the addition of 100, 300, and 500 mg/L of CQDs increased the thermal conductivity by 40, 50, and 60 %, respectively. However, the 1000 mg/L incorporated decreased the thermal conductivities of the nanofluids. The observed behavior can be attributed to the aggregate size of the nanoparticles. Furthermore, a new thermal conductivity model for CQD-based nanofluids was developed considering brine salinity, particle size distribution, and agglomeration effects. The model showed a remarkable fit with the experimental data and predicted the effect of the nanofluid concentration on the thermal conductivity and cycle efficiency. Coupled with an ORC cycle model, CQD concentrations of approximately 550 mg/L increased the cycle efficiency by approximately 13.8% and 18.6% for commercial and synthesized CQDs, respectively.

## 1. Introduction

Concerns about sustainability, energy security, affordability, greenhouse gas emissions, and climate change have increased because of high primary energy consumption, population growth, and record values of atmospheric temperatures [1]. Approximately 78% of the world’s energy consumption comes from fossil fuels [2]. While energy is still largely reliant on fossil fuels, government regulations and policies have encouraged the development and inclusion of renewable energy technologies to contribute to the energy transition. For these reasons, there is growing interest in exploring new renewable energy sources such as wind, hydropower, solar, geothermal, and tidal energy [3].

According to the International Energy Agency (IEA), geothermal energy is the energy contained in the Earth’s interior. Based on the temperature of the extracted brine, geothermal energy can be used for efficient electricity production, direct use, space heating, cooling, and other industrial applications [4]. This type of energy is presented as a promising alternative to non-renewable sources because it is clean, independent of the weather, sustainable, available, and renewable and produces less carbon dioxide, particulate matter, and other toxic substances that create the greenhouse effect [5]. Despite this significant production potential, obtaining geothermal energy compared to wind and solar energy presents some disadvantages associated with locations with high geothermal gradients, volcanic or hydrothermal activities, higher capital investments, and longer completion times due to well drilling [6]. Therefore, minimizing drilling costs is the main factor to consider in geothermal projects [7]. An alternative to the high cost of well drilling is the use of abandoned and depleted crude oil wells for geothermal development through co-production or well conversion [8,9]. Co-production is the simultaneous production of hydrocarbon and geothermal energy from a well or hydrocarbon field. Thermal energy trapped in the extracted fluid co-produced during oil and gas operations is converted into electricity and heat through the implementation of organic binary Rankine cycle (ORC) technology [7,10]. Several researchers have reported that mature oil reservoirs provide opportunities for co-production because they produce both hydrocarbons and hot formation water in the temperature range 65–150 °C [11]. The water cutoff in this reservoir type is approximately 99%, thereby lowering oil production. Thus, large volumes require expensive techniques for water separation from oil, water treatment, and disposal. Therefore, the use of thermal energy from water obtained from the co-production of oil and gas for power production represents a promising alternative. For example, Liu et al. [12] investigated the potential of harnessing low-temperature geothermal energy from hydrocarbon reservoirs using wastewater streams. The authors proposed a roadmap for screening criteria to determine the feasibility of waste heat recovery based on geological, reservoir, production, and economic parameters. The results showed that high heat recovery was associated with a high water cutoff (greater than 95%), high flow rates, a wellhead temperature of 57 °C, a geothermal gradient of at least 20 °C/km, and a highly fractured medium. Similarly, Cano et al. [8] reviewed the recent advances and applications of geothermal power systems in the oil and gas industry, starting from the fundamentals and basic principles of geothermal energy and ORC. This research investigated the use of geothermal resources from abandoned wells, active wells, and paired thermally enhanced oil recovery processes with injection for fluid heating and energy production.

Céspedes et al. [9] pioneered an assessment of the technical and environmental feasibility of co-producing oil and electric power from geothermal resources in Colombian fields. The authors reported that geothermal energy co-production systems use organic Rankine cycle engines to convert heat from reservoir production fluids into electric power. The energy potential of this resource was assessed using the exergy concept, and life cycle analysis was applied to calculate the carbon footprint using the 2013 Intergovernmental Panel on Climate Change (IPCC) methodology. The results show maximum energy production potentials of 2260 kWe and 657 kWe for the OFA and OFB fields, respectively. Regarding conventional electrical systems, previous studies indicated that the co-production of oil and electrical energy from geothermal resources could potentially reduce the carbon footprint by 19%. This study demonstrated the technical feasibility of obtaining electrical energy from geothermal resources from the oil industry in Colombia.

As mentioned previously, low-to-medium-enthalpy geothermal resources can be used for power production through the ORC. However, conventional heat transfer devices, such as heat exchangers, require operating fluids with poor thermophysical properties. This event has drawbacks in the heat transfer process, reducing the cycle efficiency. In this sense, the low heat transfer characteristics of the traditional fluids used in energy generation have led to the need to identify new technologies to improve the heat transfer process and cycle efficiency.

As nanotechnology has become increasingly popular, several nanofluids have been proposed to improve the efficiency of heat exchangers in geothermal processes [6,13]. Nanofluid properties such as density, thermal conductivity, specific heat capacity, and viscosity depend on the particle shape and size, material type and physical properties, and the amount of nanoparticles dispersed in the base liquid. In particular, the addition of metallic, metal oxide nanoparticles, or carbon-based materials to heating fluids can improve the heat transfer characteristics of fluids through changes in the heat capacity, thermal conductivity, density, and viscosity of the basic fluids. Among them, Al [14], Cu [15], Ag, [16] Au, Al_2_O_3_, [17] ZnO_2_, CuO_2_, [18] TiO_2_ [19], nanotubes, graphite, and nanodiamonds have been studied [14]. Similarly, Ag-MgO/water hybrids, Al_2_O_3_–Cu/water nanofluids, and nanofluids based on ethylene glycol and silver nanoparticles have been proposed [20].

Du et al. [18] performed a numerical simulation to evaluate the effect of the particle morphology and size on the efficiency of a geothermal heat exchanger. The results showed that CuO/water nanofluids with spherical shapes were more efficient than those with rod-shaped nanoparticles. Therefore, the authors argued that the nanoparticle morphology significantly affects the heat transfer. Jahanbin et al. [21] investigated the effects of various water-based nanofluids (Al_2_O_3_, Ag, Cu, CuO, Fe_2_O_3_, SiO_2_, and TiO_2_) on the thermal performance of U-tube heat exchangers for geothermal applications. The results showed that silver nanofluid with a 2% volume fraction showed a maximum reduction of 2.6% in the borehole thermal resistance compared to water, followed by copper nanofluid (2.5%). The silver and copper nanofluids required approximately 31% and 26% more pumping power than water, respectively. However, the aforementioned studies did not consider the presence of brine in the base of the nanofluid to determine the thermal conductivity, and the model also did not include the morphology of the nanomaterials and the content of brine in the thermal conductivity correlations.

Carbon quantum dots (CQDs) are a new class of environmentally friendly zero-dimensional (0D) nanomaterials with fluorescence characteristics [22]. Owing to their electronic and physicochemical properties, such as small size, good photostability, excellent biocompatibility, conversion, tunable photoluminescence, and chemical stability, the desired advantages of low toxicity, low cost, and simple synthetic routes, CQDs have several applications in the fields of biosensors [23], membrane technology [24], biomedicine [25], photocatalysis [26], and wastewater treatment [27,28,29].

Considering the issues of heat transfer in ORC to obtain power from the mature field, the remarkable physicochemical properties of CQDs, the renewable chemical nature, the potential low cost, and the synthesis allow CQDs to be considered an attractive alternative for the formulation of nanofluids that improve heat transfer in geothermal-based energy coproduction. However, to the best of our knowledge, no studies in the specialized literature have evaluated the addition of CQDs for geothermal applications in the process of co-production experimentally or theoretically.

Hence, the main objective of this study was to develop nanofluids based on CQDs as a heating fluid in the ORC cycle to improve heat transfer in the energy co-production process based on theoretical and experimental studies. CQD-based nanofluids with different physicochemical properties were designed for this purpose. The research included (i) the synthesis and characterization of carbon quantum dots, (ii) the study of the effects of CQD concentration on the thermal conductivity of nanofluids, and the development of a new mathematical model to predict the effect of the use of CQDs in the heating fluid of the ORC cycle for geothermal applications. This work has a remarkable impact on the oil and gas industry because of the implementation of a technology that allows the use of geothermal resources, thereby reducing the cost associated with drilling new geothermal wells. Likewise, this research contributes to the first step toward decarbonization strategies.

## 2. Materials and Methods

### 2.1. Materials

Two types of CQDs were used: (i) commercial CQDs provided by Petroraza S.A. (Sabaneta, Antioquia, Colombia) and (ii) CQDs synthesized from citric acid and ethylenediamine. Synthetic brine was composed of sodium chloride (NaCl), potassium chloride (KCl), hexahydrate magnesium chloride (MgCl_2_·6H_2_O), and calcium chloride (CaCl_2_·2H_2_O). All reagents were purchased from Sigma-Aldrich (St. Louis, MO, USA) and used without further purification. The composition of the synthetic brine is summarized in Table 1. This solution was prepared by adding the required amount of each salt to deionized water, and the solution was stirred at 600 rpm for 6 h.

### 2.2. Synthesis of Carbon Quantum Dots

CQDs were synthesized following the carbonization method assisted by microwaves proposed by Franco et al. [30]. To this end, citric acid was added to 6 mL of deionized water under constant stirring at 300 rpm for 30 min. Ethylenediamine was added dropwise as a nitrogen donor and stirred at 300 rpm for 30 min. The final mixture was placed in a microwave oven for 6 min at 300 W. The obtained solid fraction was dispersed in deionized water and filtered to remove agglomerates and unreacted materials. The order of reagent addition and slow mixing play vital roles in the synthesis owing to the exothermic nature of the reaction [31]. To ensure the dispersion of the CQD nanoparticles in the nanofluids, samples were homogenized through mechanical stirring at 300 rpm and subsequent sonication in a bath sonicator (Elmasonic E60 H, Elma, Singen, Germany) at 25 °C with a power of 500 W.

### 2.3. Characterization of Carbon Quantum Dots

Physicochemical characterization of the synthesized and commercial CQD samples was conducted to elucidate their main properties, such as density, heat capacity, size, stability, functional groups (chemical structure), and optical characteristics.

#### 2.3.1. Density

The density of CQD nanoparticles was determined at 25 °C using a standard pycnometric method. To this end, a calibrated 25 mL glass pycnometer and an analytical balance (±0.1 mg precision) were employed. Each measurement was performed in triplicate, and the results were averaged.

#### 2.3.2. The Specific Heat Capacity (Cp)

The specific heat capacity (Cp) was determined through differential scanning calorimetry (DSC), using a Q2000 calorimeter (TA Instruments, New Castle, DE, USA). Samples were sealed in aluminium pans and heated from 20 to 80 °C at a rate of 10 °C/min under a nitrogen atmosphere. Sapphire was used as the calibration standard. Three replicates were conducted for each sample, and the standard deviation remained below 3%.

#### 2.3.3. Fourier Transform Infrared Spectroscopy (FTIR) Analysis

Fourier Transform Infrared Spectroscopy (FTIR) was used to determine the functional groups in the CQD structure. The FTIR spectra were obtained with an IRAffinity-1S spectrophotometer (Shimadzu, Torrance, CA, USA) in the 4000 and 400 cm^−1^ range, using the methodology reported in a previous study [32].

#### 2.3.4. Particle Size and Surface Charge

The CQDs were characterized by their hydrodynamic size (dp50) using dynamic light scattering (DLS). The nanomaterials were dispersed in an aqueous phase and measured using NanoPlus-3 (Micromeritics, Norcross, GA, USA). Similarly, the zeta potentials of the CQDs were measured under synthetic brine conditions using Nanoplus-3. This analysis enabled the identification of the particle stability based on the surface charges of the CQDs. The tests were performed in triplicate to ensure measurement accuracy.

#### 2.3.5. Fluorescence Measurements

The fluorescence spectra of the commercial and synthesized CQDs were obtained using a PerkinElmer LS-55 spectrofluorometer (Waltham, Waltham, MA, USA). Dispersions containing 100 mg/L of each CQD were prepared using distilled water. The dispersions were placed in a spectrofluorometer to determine the maximum excitation and emission wavelengths.

#### 2.3.6. UV–VIS Absorption

CQD’s optical properties of the CQDs were evaluated using a Genesys 10S UV-Vis spectrophotometer Thermo Scientific, Waltham, MA, USA). Assays were conducted for each nanomaterial in an aqueous solution at 100 mg/L in the 200–600 nm UV–VIS range.

### 2.4. Nanofluid Formulations

Several nanofluids were prepared using both commercial and synthesized carbon quantum dots (CQDs) dispersed in synthetic brine. For each formulation, 200 mL of synthetic brine was used as the base fluid. CQDs were added at concentrations of 0, 100, 300, 500, and 1000 mg/L. The mixtures were magnetically stirred at 600 rpm for 6 h at room temperature, followed by sonication in an ultrasonic water bath (Elmasonic E60 H, Elma, Singen, Germany) for 2 h at 25 °C to ensure a uniform dispersion. The colloidal stability of the nanofluids was evaluated over 24 h. Zeta potential measurements were conducted to assess electrostatic stability, while UV–Vis spectroscopy was used to monitor potential sedimentation or aggregation over time.

### 2.5. Thermal Conductivity

The thermal conductivity of the nanofluids was determined using a TEMPOS analyzer (METER, München, Germany). The nanofluids were placed in a cell in contact with the sensor and were maintained at 20 °C for 12 h during the measurements. The assays were repeated six times to ensure the reproducibility of measurements.

## 3. Simulation of the Use of CQDs in Brine as Heating Fluid for ORC Cycles

As mentioned before, one of the main uses of nanofluids is in heat-transfer processes because a low nanoparticle concentration can significantly increase the thermal conductivity of the base fluid [33,34]. Therefore, the aim of this section is to assess the feasibility of using CQDs in brine as a heating fluid during low-temperature ORC cycling. For this purpose, a mathematical model for the heat transfer in an ORC cycle evaporator was developed.

The ORCmKit tool in Python versión 6.5.0 was used to model the ORC [35]. The properties of the working fluid were calculated using the CoolProp library versión 6.5.0 [36], which is a freely available software tool that is similar to the NIST RefProp library. The ORC cycle efficiency was computed as follows:

(1) $$\eta_{cycle}=\frac{{\dot{W}}_{turbine}-{\dot{W}}_{pump_{ORC}}-{\dot{W}}_{pump_{EV}}}{{\dot{Q}}_{H}}$$

where ${\dot{Q}}_{H}$ (W) is the heat transferred to the evaporator, ${\dot{W}}_{pump_{EV}}$ (W) is the consumed power by the pumping system of the evaporator circuit on the hot-fluid side, and ${\dot{W}}_{pump_{ORC}}$ (W) is the consumed pumping power to move the working fluid within the power cycle. ${\dot{Q}}_{H}$ (W) is the logarithmic mean temperature difference.

(2) $${\dot{Q}}_{H}=UA{∆T}_{ML}$$

where A (m^2^) is the surface area of the evaporator, ${∆T}_{ML}$ (K) is the logarithmic mean temperature difference, and U (W m^−2^ K^−1^) is the overall heat transfer coefficient:

(3) $$U=\frac{1}{R_{eq}A}$$

The equivalent thermal resistance in the evaporator $R_{eq}$ is expressed as

(4) $$R_{eq}=\frac{1}{hA}+\sum_{i}R_{i}$$

where $R_{i}$ is the i-th thermal resistance in addition to the convection on the side of the hot fluid. The convection heat transfer coefficient of the hot fluid side h (W m^−2^ K^−1^) can be calculated using the widely used Dittus–Boelter correlation, as follows:

(5) $$Nu=\frac{hD}{k_{nf}}=0.027{Re}^{0.8}{Pr}^{0.3}$$

where $k_{nf}\;$ (W m^−1^ K^−1^) is the thermal conductivity of the nanofluid, D  (m) is the inner diameter of the pipe comprising the evaporator, Re is the Reynolds number, and Pr  is the Prandtl number. The required pumping power was calculated from the volumetric flow and pressure drop in the evaporator.

(6) $${{\dot{W}}^{\prime }}_{pump_{HS}}\left[\frac{W}{m}\right]=-\dot{V}\frac{\partial P}{\partial x}=\dot{V}f\rho_{nf}\frac{u^{2}}{2D}$$

The friction factor of the evaporator, f, is calculated using the Colebrook–White correlation, as follows:

(7) $$\frac{1}{\sqrt{f}}=-2\times \;log\left(\frac{\frac{\epsilon }{D}}{3.7}+\frac{2.51}{Re\sqrt{f}}\right)$$

where ϵ is the rugosity of the pipe on the side of the hot fluid. To accurately calculate the Nusselt number, a comprehensive understanding of thermal conductivity is required. Given the complex nature of nanofluids, this parameter is influenced by multiple factors, including the thermal conductivity of the base fluid and nanoparticles, temperature, nanoparticle sphericity, viscosity, and volumetric fraction of nanoparticles [37]. To address this intricate interdependency, several authors have proposed empirical correlations aimed at predicting the effective thermal conductivity of nanofluids. One seminal correlation, originating from the early literature, is as follows:

(8) $$\frac{k_{nf}}{k_{f}}=\frac{k_{p}+2k_{f}-2\phi \left(k_{f}-k_{p}\right)}{k_{p}+2k_{f}+\phi \left(k_{f}-k_{p}\right)}$$

where $k_{p}$ (W m^−1^ K^−1^) is the thermal conductivity of the nanoparticles,$\;k_{f}$ (W m^−1^ K^−1^) is the thermal conductivity of the base fluid, and ϕ is the volumetric fraction of nanoparticles in the nanofluid. One of the main limitations of Equation (7) is that it does not consider the particle sphericity. Therefore, [38], we developed the following expression:

(9) $$\frac{k_{nf}}{k_{f}}=\frac{k_{p}+\left(n-1\right)k_{f}-\left(n-1\right)\phi \left(k_{f}-k_{p}\right)}{k_{p}+\left(n-1\right)k_{f}+\phi \left(k_{f}-k_{p}\right)}$$

where the parameter n is given by

(10) $$n=\frac{3}{\psi }$$

where ψ is the sphericity calculated as follows:

(11) $$\psi =\frac{Surface\;area}{surface\;area\;of\;a\;sphere\;with\;the\;same\;volume}$$

However, the thermal conductivities of some nanofluids are higher than those of the base fluid and nanoparticle material [39]. This behavior can be attributed to the Brownian motion promoted by the nanoparticles as a result of the temperature effect. In this regard [40], Equation (8) is corrected by including a Brownian motion term, as shown in the following equation:

(12) $$\frac{k_{nf}}{k_{f}}=\frac{k_{p}+\left(n-1\right)k_{f}-\left(n-1\right)\phi \left(k_{f}-k_{p}\right)}{k_{p}+\left(n-1\right)k_{f}+\phi \left(k_{f}-k_{p}\right)}+\frac{\rho_{p}\phi c_{p}}{2k_{f}}\sqrt{\frac{2k_{B}T}{3\pi d_{p}\mu }}$$

where ***dp*** (m) is the particle size, ***ρp*** (kg m^−3^) is the density of the nanoparticles, $c_{p}$ (J kg^−1^ K^−1^) is the heat capacity of the nanofluid, $k_{B}$ is the Boltzmann constant, and ***μ*** (Pa s) is the viscosity of the nanofluid. Thus, Equation (11) can be considered the most widely accepted correlation for modeling the effective thermal conductivity of nanofluids. Additionally, the other semiempirical equations presented in Table 2 were tested to determine which model fits best in this specific case.

## 4. Results and Discussion

### 4.1. Characterization of CQDs

**Figure 1:**
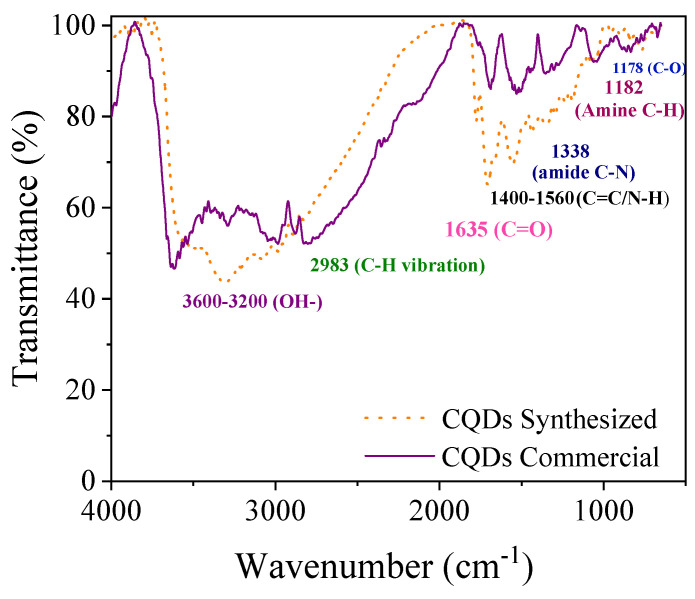
Comparative FTIR spectra of commercial and synthesized CQDs.

Table 3 lists the physicochemical properties of the CQDs. Commercial CQDs have a higher density and heat capacity than synthesized CQDs. The FTIR spectra of the commercial and synthesized CQDs are shown in Figure 1. Both spectra present similar peaks, which are in accordance with those reported previously [29,30]. In general, the highlighted bands were associated with carbonaceous bonds and the presence of nitrogen groups, which were obtained from the ethylenediamine source. The wide band around 3600–3200 cm^−1^ corresponds to the stretching vibration of O–H. The peak at 2983 cm^−1^ is associated with the C–H vibration. The band at approximately 1338 cm^−1^ represents the presence of amide C–N bonds. The band corresponding to amine C–H stretching bonds appeared at 1182 cm^−1^. The signals at 1635 and 1178 cm^−1^ correspond to C=O and C–O stretching vibrations, respectively. The peak at approximately 1400–1560 cm^−1^ is associated with the bending vibration of C=C/N–H and C–N stretching. The peak at 1570 cm^−1^ is attributed to the bending vibrations of N-H. The representative bands were in accordance with those reported previously [29,30,46].

**Figure 2:**
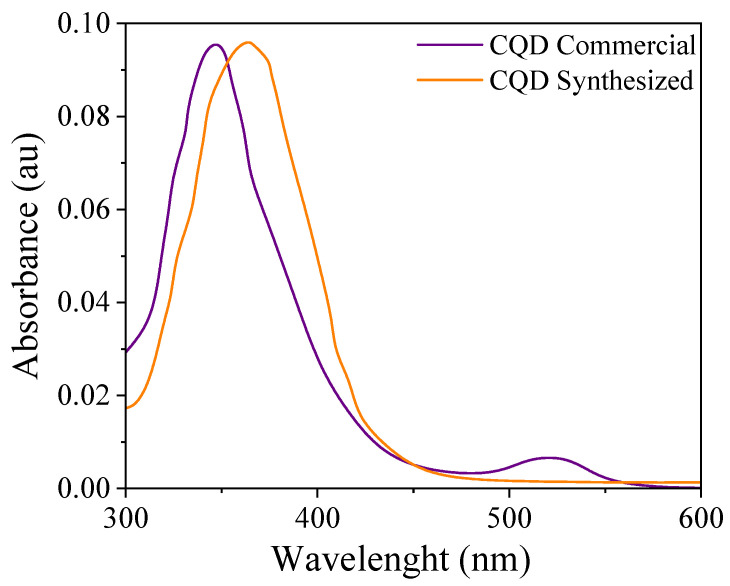
UV–VIS absorption spectra of CQD nanomaterials.

Figure 2 shows the UV–VIS absorption spectra of the nanomaterials. It can be observed that both CQDs absorb energy within the same range. The peak at approximately 300 nm can be related to the n-pi transition of the C=O bonds or other linked groups present on the CQD surface. This result is consistent with that reported by Mandal et al. [47].

**Figure 3:**
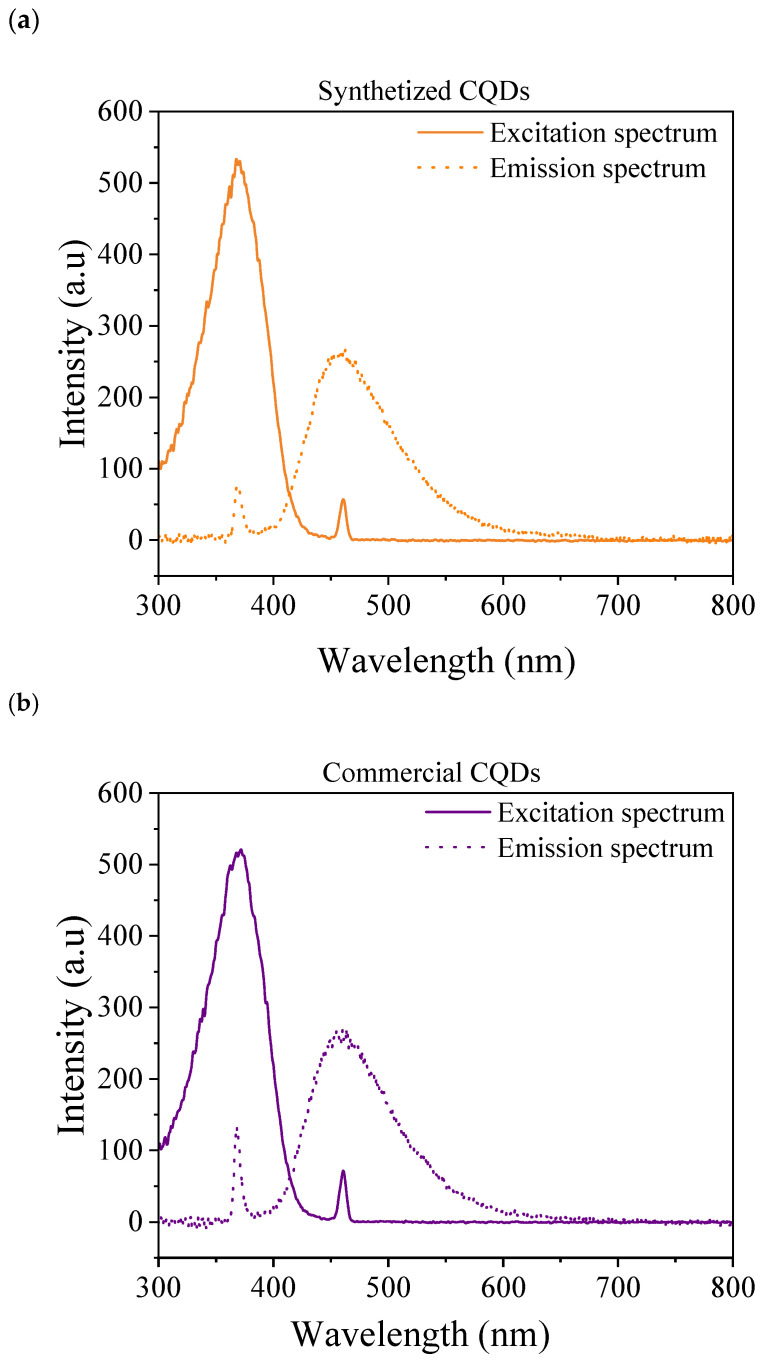
Fluorescence excitation and emission spectrum: (**a**) synthesized CQDs and (**b**) commercial CQDs.

The excitation and emission spectra of the synthesized and commercial CQDs are shown in Figure 3. The results show that both CQDs exhibit the same excitation and emission spectra. The maximum excitation–emission signals were 368–460 nm for both CQDs. Considering the classification based on emission length, the analyzed CQDs were in the blue range [48].

**Figure 4:**
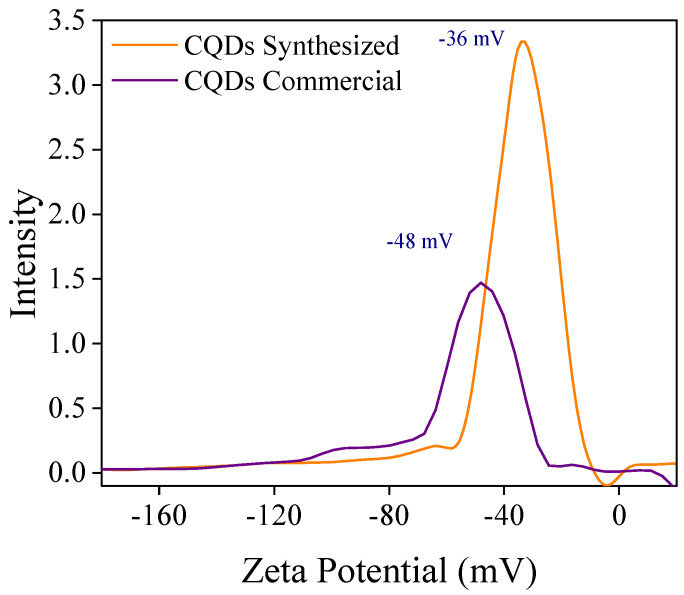
Z potential of CQD nanomaterials.

Figure 4 shows the distribution of the zeta potentials of the CQD. The results indicate that both nanomaterials have a negative surface charge, which is more noticeable in commercial CQDs. This difference can be attributed to the type of reagent used in the initial synthesis. Based on a previous report, a z-potential measurement of |25 mV| indicated that the colloidal suspension was stable. In this study, a stable suspension was required to increase the conduction and heat transfer through the convention. This agrees with the report by Razzaq et al. [34], who evaluated the effect of the colloidal stability of nanofluids on the heat transfer process. It is important to note that the z potential is an important parameter in the colloidal stability of nanofluids [49].

**Figure 5:**
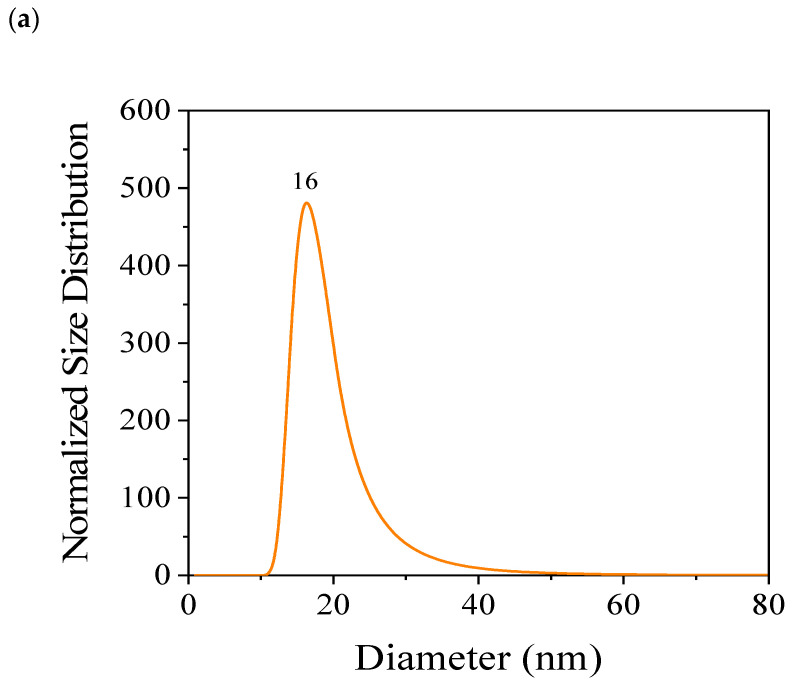

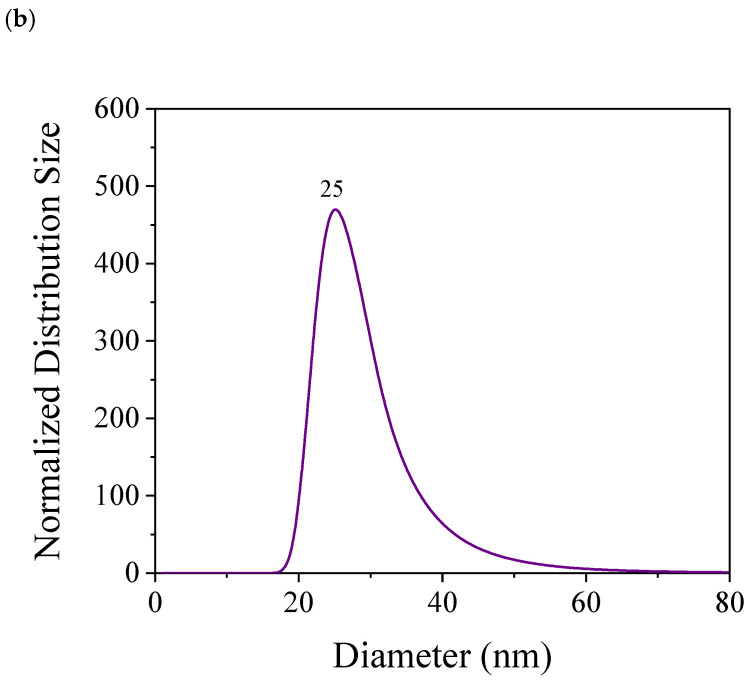
Size distribution of CQD nanoparticles measured through DLS; (**a**) synthesized and (**b**) commercial CQDs.

Figure 5 shows the size distribution of the CQD nanoparticles. The results indicate a narrow size distribution, revealing an average particle size of approximately 16 and 25 nm for in-house and commercial CQDs, respectively. The differences in the size of CQDs are associated with the synthesis method employed for CQD production, described by Quaid et al. [25], who studied the effects of the synthesis process, temperature, and reaction time on the chemical, morphological, and quantum properties of CQDs obtained from Loblolly Pine. The particle size determined corresponds to the particle size of the nanomaterial dispersed in the fluid.

### 4.2. Thermal Conductivity

The stability of the synthesized and commercial CQDs was monitored through visual inspection, as well as through absorbance and fluorescence measurements for one week. No significant changes were observed, with ±1% variation in the absorbance and fluorescence measurements and no visual precipitation or destabilization of both samples. This is in agreement with the results reported by Franco et al. [30], who measured the stability of CQDs at different temperatures in brine solutions for up to 5 months and found that fluorescence did not change significantly, even when samples were exposed to sunlight.

**Figure 6:**
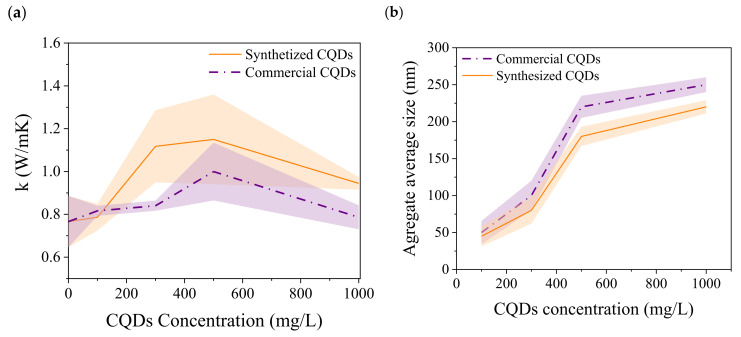
Effect of nanoparticle concentration on the thermal conductivity of nanofluids (**a**); effect of nanoparticle concentration on the average aggregate size (**b**). The uncertainty of measurements was ±0.063.

The effect of the CQD concentration on the thermal conductivity of the nanofluids is shown in Figure 6. The thermal conductivity of the nanofluids increased as the CQD concentration increased from 100 to 500 mg/L. Then, it is observed that from 500 mg/L, the conductivity decreased. This effect was more noticeable in fluids based on the synthesized CQDs. Several researchers have reported an increase in thermal conductivity with an increase in nanoparticle concentration or volume fraction, which [13,49] is related to the Brownian motion of the particles and thermophoresis caused by a temperature gradient in the fluid. Sometimes, thermophoresis can have a larger effect than Brownian motion [50]. However, both factors contributed to increased particle collisions, thereby improving the thermal conductivity. On the other hand, Brownian motion can cause the stirring of water molecules, which creates microconvection within the nanofluid, thereby increasing heat transfer [37].

The decrease in thermal conductivity from 500 mg/L to 1000 mg/L can be related to the aggregation of nanoparticles, which reduces the surface area and possible surface interactions. To clarify this observation, we evaluated the average aggregate size of the nanoparticles at the studied concentrations (Figure 6b). The results suggest that the average aggregate size increased significantly at 1000 mg/L CQDs. The average aggregate size is more noticeable for nanofluids based on commercial CQDs. As expected, the average aggregate size affected the thermal conductivity reduction. These results agree with those reported by [51], who studied the average diameter of particle agglomerates using the dynamic light scattering method. The authors observed changes in the agglomerates over time and the concentration of the nanoparticles. 

On the other hand, the size of the nanoparticles significantly affected the nanofluid thermal conductivity. As the nanoparticle size increased, the thermal conductivity of the nanofluid decreased. This can be explained by the Brownian effect of particle size on the Brownian effect [52]. Larger particles have lower Brownian motion, indicating that the movement of the nanoparticles in the base fluid is less. Smaller particles can move faster and have greater Brownian motion, thereby creating more convection in the base fluid. Additionally, nanoparticles can carry energy because of heat transfer, which depends on several factors, including the size and velocity of the nanoparticles. Diffusive heat transfer allows the heat absorbed by particles to be carried to other locations throughout the base fluid [37].

In this case, the base fluid was brine containing several salts (calcium, magnesium, potassium, and sodium chloride). The thermal conductivity of the brine was 0.766 W/mK, and that of the ionized water was 0.63 W/mK. These results indicate that the presence of salts affected the thermal conductivity of the fluid. For this reason, CQD-based nanofluids increase thermal conductivity because of the possible surface interactions between the functional groups of the CQDs and the ionic presence in the salts.

As expected, the thermal conductivity depends mainly on the type of nanoparticle (size, morphology, and chemical structure) and the particle concentration present in the nanofluid [49,53]. However, other thermal and relevant non-thermal factors, such as density, heat capacity, base fluid, and medium temperature, flow rate, pressure drop, particle shape and size, viscosity, and Brownian motion, can affect these properties [34]. Likewise, the base of the fluid has a significant effect on the thermal conductivity due to possible surface interactions with the nanomaterial.

**Figure 7:**
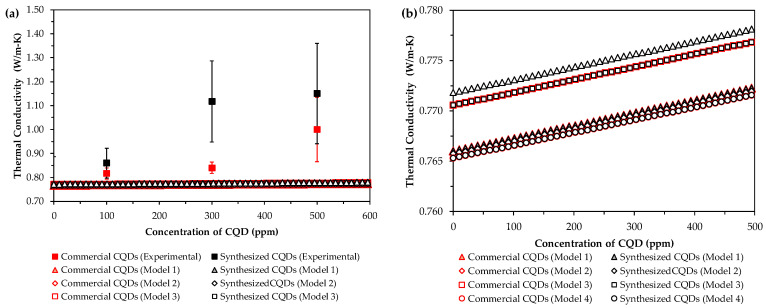
Comparison of effective thermal conductivity models with experimental Data. Models 1–5 correspond to the correlations listed in Table 2. Figure (**b**) represents the models zoom of Figure (**a**).

The fits of the five evaluated models to the experimental thermal conductivity data are shown in Figure 7. All the models underestimated the experimental data, which can be attributed to the fact that the evaluated models are strictly valid only for pure liquids mixed with nanoparticles. It is worth noting that this study considers the ions present in the brine that interact with the CQDs and water, thereby altering the thermal conductivity of the nanofluid. Additionally, other authors have reported atypical increases in the thermal conductivities of CQD-fabricated nanofluids. For example, Chen et al. [54] reported an increase of 43.2% in conductivity with 0.1 vol% CQDs, and Mousavi et al. [55] reported an increase of approximately 25% in thermal conductivity with 0.5 vol% CQDs. This implies that the effect of CQDs on conductivity is not fully understood. Therefore, conventional nanofluid conductivity models cannot adequately predict the behaviors of the nanofluids investigated in this work.

Based on the above results, a modified model was developed to account for the effect of salinity on the thermal conductivity of the nanofluid. To this end, the effective conductivity of the new fluid was expressed as a composite function between Equation (12) and the salinity of brine,$\;S\left[g/l\right].\;$ This composite function is shown below, where $g1\left(S,\phi \right)$, $g2\left(S,\phi \right)$, $g3\left(S,\phi \right)$, $g4\left(S,\phi \right),g5\left(S,\phi \right),$ and $g6\left(S,\phi \right)$ are salinity functions of linear, polynomial, exponential, and potential types, respectively.

(13) $$\begin{array}{c} k_{nf}=g1\left(S,\phi \right){\left[\frac{k_{p}+\left(n-1\right)k_{f}-\left(n-1\right)\phi \left(k_{f}-k_{p}\right)}{k_{p}+\left(n-1\right)k_{f}+\phi \left(k_{f}-k_{p}\right)}\right]}^{g2\left(S,\phi \right)}\\ \;\;\;\;\;\;\;\;\;\;\;\;\;\;\;\;\;\;\;\;\;\;\;\;\;\;\;\;\;\;\;\;\;\;\;\;\;+\int_{0}^{\infty }g3\left(S,\phi \right){\left[\frac{\rho_{p}\phi^{g4\left(S,\phi \right)}\phi c_{p}}{2k_{f}}\sqrt{\frac{2k_{B}T}{3\pi d_{p}\mu }}\right]}^{g5\left(S,\phi \right)}f\left(d_{p}\right)dd_{p}\\ \;\;\;\;\;\;\;\;\;\;\;\;\;\;\;\;\;\;\;\;\;\;\;\;\;\;\;\;\;\;\;\;\;\;\;\;\;+g6(S,\phi ) \end{array}$$

It is important to note that the second term on the RHS (Equation (13)) is modified with an integral, which is performed to account for the effect of non-uniformity in the nanoparticle size distribution $f\left(d_{p}\right)$. In contrast, Equation (12) is strictly valid for uniform particle-size distributions. To determine the best fit, the criterion was the highest goodness-of-fit statistic (the adjusted R-squared value. This statistic is generally the best indicator of fit quality when comparing nested models, that is, a series of models, each of which adds additional coefficients to the previous model [56]. In other words, the adjusted R-squared value penalizes the unnecessary inclusion of constants in the fitting model. Using MATLAB 2023b’s Optimization Toolbox, 17,260 fitting function options were evaluated. Finally, the best adjusted R-squared value was obtained using the following expression:

(14) $$\begin{array}{c} \frac{k_{nf}}{k_{f}}=\frac{k_{p}+\left(n-1\right)k_{f}-\left(n-1\right)\phi \left(k_{f}-k_{p}\right)}{k_{p}+\left(n-1\right)k_{f}+\phi \left(k_{f}-k_{p}\right)}\\ \;\;\;\;\;\;\;\;+\int_{0}^{\infty }\beta_{1}S\frac{\rho_{p}\phi^{\beta_{2}S}c_{p}}{2k_{f}}\sqrt{\frac{2k_{B}T}{3\pi d_{p}\mu }}f\left(d_{p}\right)d_{p} \end{array}$$

where $\beta_{1}=0.049l/g$ and $\beta_{2}=0.025l/g$ are constants.

**Figure 8:**
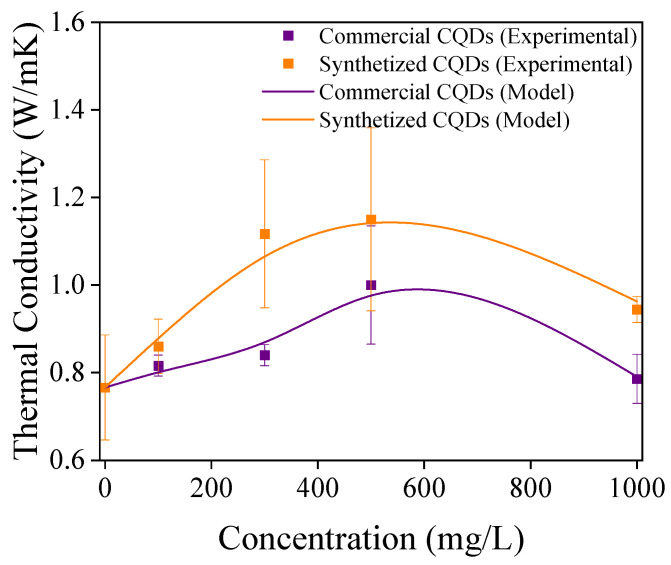
Predictions of the modified thermal conductivity model.

**Figure 9:**
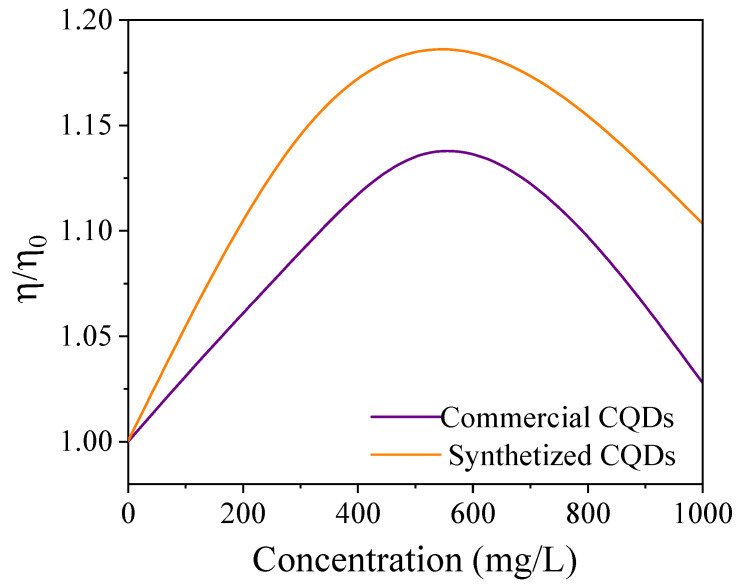
Prediction of ORC cycle efficiency as a function of CQD concentration in the nanofluid ($\eta_{0}=1.077\%)$.

Figure 8 shows the proper adjustment of the experimental data using the modified model. The proposed model enables the estimation of adjustable parameters within realistic ranges. Similarly, sphericity values of 0.45 and 0.49 for commercial and synthesized CQDs, respectively, were obtained. These values are typical of cylinders with a negligible aspect ratio [57], which is consistent with the flake-like structure found in the morphological studies of CQDs [29,30]. The calculated thermal conductivities of the nanoparticles were 6.5 and 6.8 W/mK for commercial and synthesized CQDs, respectively. These results are within the typical range of magnitudes of nanostructured carbonaceous materials [58]. Although the proposed model is a semi-empirical approximation, given the correlation form, it is inferred that the main effect of the species present in the brine is to enhance the Brownian motion of the nanoparticles. Furthermore, Figure 9 shows that the proposed model is capable of capturing the effect of aggregation on the thermal conductivity of the nanofluid, predicting a maximum conductivity value at a concentration of approximately 560 mg/L. This is because as the concentration of CQDs increases, the average aggregate size also increases (see Figure 6b), leading to a decrease in Brownian motion agitation.

The ORC cycle efficiency was predicted as a function of the CQD concentration in the nanofluid (Figure 9). As expected, an increase in the CQD concentration increases the overall efficiency of the ORC cycle, reaching maximum efficiency values that exceed those of the initial cycle: $\eta_{0}$ by approximately 13.8% for commercial CQDs and approximately 18.6% for synthesized CQDs. Interestingly, the two maximum calculated values occurred at nearly identical CQD concentrations of approximately 550 mg/L. Thus, this finding demonstrates the relationship between the CQD concentration and the ORC cycle efficiency. These results support the feasibility and potential of CQDs as performance enhancers in thermal energy generation applications.

**Figure 10:**
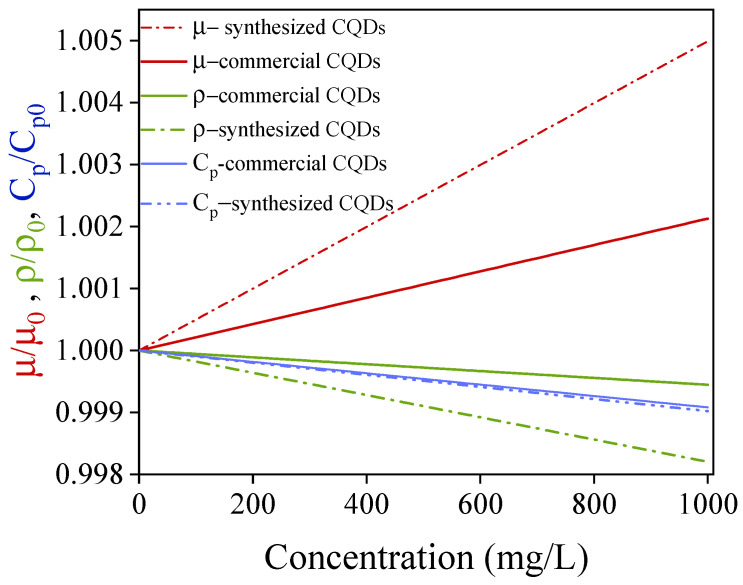
Prediction of the viscosity, density, and specific heat variation in nanofluids with different concentrations of CQDs.

Based on the previous analysis, low CQD concentrations can lead to significant increases in the thermal conductivity of the nanofluid, thereby improving the ORC cycle efficiency. However, the inclusion of nanoparticles may adversely affect the other functional properties of the fluid. Therefore, the effect of CQD on the viscosity, density, and specific heat of the nanofluid was also evaluated. Figure 10 shows the effect of CQD concentration on the functional properties of the nanofluids. Minor changes of less than 1% can be observed for the viscosity, density, and specific heat of nanofluids at several CQD concentrations. This result implies that significant increases in the pumping costs or energy storage capacity of the nanofluid are not expected. Therefore, fluids produced using CQDs in brines are a highly attractive alternative for use in heat exchange equipment. The implemented model is based on the modification of Maxwell’s classical conductivity equation, considering important parameters such as Brownian movement, volumetric fraction, the particle size distribution, and the base fluid (brine). However, the model limitations are associated with the constant B obtained from the adjustment parameters. Thus, this constant does not have any physical significance, and they can adopt other values or inclusive to be function.

## 5. Conclusions

Two types of CQD nanoparticles were used as additives in a heating fluid to enhance heat transfer in the coproduction of heat and hydrocarbons for geothermal applications. The CQD nanoparticles were obtained by the synthesis of citric acid and ethylenediamine. The nanomaterials were characterized in terms of their density, morphology, functional groups, zeta potential, and heat capacity. The results showed that the synthesized CQDs had similar chemical structures and UV absorption spectra. In contrast, the commercial CQDs exhibited higher size, zeta potential, density, and heat capacity than the synthesized CQDs. The developed nanofluids exhibited high thermal conductivity, which was more noticeable in nanofluids containing the synthesized CQDs. These results are associated with differences in the physicochemical properties and surface interactions between CQDs and brine salts.

A new thermal conductivity model for CQD-based nanofluids was developed considering the effects of brine salinity, particle size distribution, and nanoparticle agglomeration. The model showed a remarkable fit with the experimental data and enabled the prediction of a decrease in the thermal conductivity of the nanofluid at 1000 mg/L. This behavior can be attributed to the surface interactions between the functional groups of the CQDs and the salts in the brine.

The developed thermal conductivity model was coupled to the ORC cycle model ORCKit and the CoolProp libraries of Python. It was estimated that when the CQDS concentration was 550 ppm, the cycle efficiency increased by approximately 13.8% for commercial CQDs and 18.6% for in-house synthesized CQDs.

It was estimated by the model that changes in properties such as viscosity, density, and heat capacity of nanofluids are negligible owing to the low concentration of CQDs required to increase the conductivity of the heating fluid. It can be observed that minor changes of less than 1% for the viscosity, density, and specific heat of nanofluids occur as the concentrations of CQDs increase.

The proposed model predicted the nonlinear dependence of thermal conductivity on CQD concentration under experimentally stable conditions. Unlike conventional models, this correlation accounts for brine salinity, particle aggregation effects, and particle size distribution, factors particularly relevant to the behavior of CQDs in realistic geothermal fluids. However, the model limitations are associated with β constants, which do not have a physical significance and are constant for this case. Specifically, it would require varying the system conditions further to determine whether these constants take on other values or can be functions.

It was expected that the use of CQD-based nanofluids in geothermal approaches could be extended to secondary fields in wells, for example, in Enhanced Oil Recovery (EOR) technology.

## Figures and Tables

**Table 1:** Brine content in the nanofluid formulation.

Component	Concentration (g/L)
Sodium Chloride (NaCl)	6.82
Potassium chloride (KCl)	0.093
Hexahydrate magnesium chloride (MgCl_2_6H_2_O)	0.634
Calcium chloride dehydrated (CaCl_2_2H_2_O)	0.543

**Table 2:** List of correlations used for the thermal conductivity calculations in this study.

Number	Model	Reference
1	$k_{nf}=\left[\frac{k_{p}+\left(n-1\right)k_{f}-\left(n-1\right)\phi \left(k_{f}-k_{p}\right)}{k_{p}+\left(n-1\right)k_{f}+\phi \left(k_{f}-k_{p}\right)}+\frac{\rho_{p}\phi c_{p}}{2k_{f}}\sqrt{\frac{2k_{B}T}{3\pi d_{p}\mu }}\right]k_{f}$	[41]
2	$k_{nf}=\left(1-\phi \right)k_{f}+\beta_{k}k_{p}\phi +18\times \;10^{6}\frac{d_{f}}{d_{p}}k_{f}{\left(\frac{k_{B}T}{3\pi \mu_{p}l_{f}}\right)}^{2}{Pr}_{\phi_{p}}$	[42]
3	$k_{nf}=k_{f}\left[\frac{k_{p}+2k_{f}-2\phi \left(k_{f}-k_{p}\right)}{k_{p}+2k_{f}+\phi \left(k_{f}-k_{p}\right)}\right]+\frac{\varsigma \lambda_{p}C_{p}}{3}\frac{k_{B}T}{3\pi \mu d_{p}\lambda_{f}}\left[1+\frac{3\pi \mu d_{p}\lambda_{f}\sqrt{Aq^{2}\lambda_{p}}}{k_{B}T\sqrt{\varepsilon_{f}m_{p}{\left({\frac{d_{p}}{2}\phi }^{-1/3}\right)}^{2}}}\right]$	[43]
4	$k_{nf}=\left(1+\frac{2k_{B}T}{\pi \mu {d_{p}}^{2}}\times C\frac{\phi d_{f}}{k_{f}\left(1-\phi \right)d_{p}}\right)k_{f}\;\;\;\;\;\;\;\;$ where $C=\psi \lambda_{p}nk_{B}$	[44]
5	$k_{nf}=\frac{k_{p}+2k_{lr}+2\left(k_{p}-k_{lr}\right)\left(\frac{d_{p}}{d_{p}+2\delta }\right)}{k_{p}+2k_{lr}-2\left(k_{p}-k_{lr}\right)\left(\frac{d_{p}}{d_{p}+2\delta }\right)}\left(\frac{2k_{lr}}{d_{p}+2\delta }\right)$	[45]

**Table 3:** Properties of the employed CQDs.

CQDs	Density ± 0.012 (g/cm^3^)	Heat Capacity ± 0.017 (J/g·°C)
Commercial	1.175	0.350
Synthesized	0.501	0.100

## Data Availability

The original contributions presented in the study are included in the article, further inquiries can be directed to the corresponding author/s.

## List of Symbols

$\eta_{cycle}$	Cycle efficiency.
${\dot{Q}}_{H}$	Heat transferred to the evaporator
${\dot{W}}_{pump_{EV}}$	Power consumed by the pumping system of the evaporator circuit on the hot-fluid side.
${\dot{W}}_{pump_{ORC}}$	Pumping power consumed to move the working fluid within the power cycle.
A	Surface area of the evaporator
${∆T}_{ML}$	Logarithmic mean temperature difference.
U	Overall heat-transfer coefficient
$R_{eq}$	Resistance in the evaporator
$R_{i}$	i-th thermal resistance
h	Convection heat transfer coefficient of the hot fluid side
Nu	Nusselt number
Re	Reynolds number
Pr	Prandtl number
$k_{nf}$	Thermal conductivity of nanofluid
D (m)	Inner diameter of the pipe that comprises the evaporator
f	Friction factor in the evaporator
ϵ	Rugosity
$k_{p}$	Thermal conductivity of the nanoparticles,
$k_{f}$	Thermal conductivity of the base fluid
ϕ	Volumetric fraction of nanoparticles in the nanofluid.
ψ	Sphericity
n	Sphericity parameter
$d_{p}$	The particle size
$\rho_{p}$	Density of nanoparticles
$c_{p}$	Heat capacity of nanofluid
$k_{B}$	Boltzmann constant
μ	Viscosity of the nanofluid.
